# Implementation of a net benefit parameter in ROC curve decision thresholds for AI-powered mammography screening

**DOI:** 10.3389/fdata.2025.1690955

**Published:** 2025-11-18

**Authors:** Anastasia Petrovna Pamova, Yuriy Aleksandrovich Vasilev, Tatyana Mikhaylovna Bobrovskaya, Anton Vyacheslavovich Vladzimirskyy, Olga Vasilyevna Omelyanskaya, Kirill Mikhailovich Arzamasov

**Affiliations:** 1 Research and Practical Clinical Center for Diagnostics and Telemedicine Technologies of the Moscow Health Care Department, Moscow, Russia; 2 Pirogov National Medical and Surgical Center, Moscow, Russia; 3 I.M. Sechenov First Moscow State Medical University of the Ministry of Health of the Russian Federation, Moccow, Russia; 4 RTU MIREA - Russian Technological University, Moscow, Russia

**Keywords:** medical care, population health, artificial intelligence, screening, machine learning, mammography

## Abstract

**Background:**

The rapid integration of artificial intelligence (AI) into mammography necessitates robust quality control methods. The lack of standardized methods for establishing decision thresholds on the Receiver Operating Characteristic (ROC) curves makes it challenging to judge the AI performance. This study aims to develop a method for determining the decision threshold for AI in screening mammography to ensure the widest possible population of women with a breast pathology is diagnosed.

**Methods:**

Three AI models were retrospectively evaluated using a dataset of digital mammograms. The dataset consisted of screening mammography examinations obtained from 663,606 patients over the age of 40. Our method estimates the decision threshold using a novel approach to net benefit (NB) analysis. Our approach to setting the cutoff threshold was compared with the threshold determined by Youden's index using McNemar's test.

**Results:**

Replacing the Youden index with our method across three AI models, resulted in a threefold reduction in false-positive rates, twofold reduction in false-negative rates, and twofold increase in true-positive rates. Thus, the sensitivity at the cutoff threshold determined by NB increased to 99% (maximum) compared to the sensitivity determined by Youden's index threshold (72% maximum). Correspondingly, the specificity when using our method decreased to 48% (minimum), compared to 75% (minimum) with the Youden's index method.

**Conclusions:**

We propose using AI as the initial reader together with our novel method for determining the decision threshold in screening with double reading. This approach enhances the AI sensitivity and improves timely breast cancer diagnosis.

## Introduction

The healthcare systems face increasing burden and personnel shortages, leading to elevated rates of medical errors. Routine tasks contribute to professional burnout, diminishing the quality of care. To address these challenges, AI technologies are being actively integrated into healthcare, aiming to improve care quality (Vasilev et al., 2023a,b,c). AI applications range from screening, diagnosis, and prognosis of health outcomes to the creation of medical decision support systems, and healthcare optimization (Liu et al., 2021).

One of the key areas for AI application in healthcare is breast screening (mammography) (Morozov et al., 2020a,b). Breast cancer is a leading cause of death among women, accounting for over 19.1% of all cancers in Russia (Huang et al., 2021). The main screening methods are digital and analog mammography (MMG) (Evans et al., 2013). However, a radiologist is not always able to accurately interpret MMG results and provide timely referrals for histological examination (Bae et al., 2014). This increases the time it takes for diagnosis establishment and treatment initiation (missed breast cancers diagnoses due to human error can reach 31%) (Hovda et al., 2023). One way to address these issues is using AI (Vasilev et al., 2023a, p. 10). While it seems simple, in clinical practice turning an AI into a reliable pathology detector may be challenging (Olczak et al., 2021; Hicks et al., 2022). The bottlenecks include dataset labeling, selecting metrics for evaluating AI performance, choosing a decision threshold for binary classification (including screening MMG), and other challenges.

Developers working with computer vision and binary classification utilize ROC curves to evaluate AI performance. The ROC curve is a graphical plot that illustrates the performance of a classification model at all possible threshold values. ROC analysis employs quantitative metrics such as specificity, sensitivity, accuracy, F-1 score, to optimize AI performance across classification thresholds. AI's high sensitivity becomes crucial when a disease or pathology is contagious or associated with serious complications. In this scenario, incorrect classification may cause severe consequences, such as spread of infection or patient's death. Other scenarios benefit from high specificity, such as incorrectly classifying a finding as pathological followed by risky and expensive diagnostic tests, such as biopsy (Habibzadeh et al., 2016; Cho et al., 2021). To optimize the AI fine tuning for digital MMG we used ROC analysis, a widely accepted and beneficial method in healthcare. To that end, it was necessary to choose an optimal decision threshold that would fit the AI's task (Liu et al., 2019).

The ROC curve visualizes the binary classification of an AI model across various thresholds. It plots the trade-off between sensitivity (the model's ability to correctly identify positive cases) and specificity (its ability to correctly identify negative cases) as the decision threshold changes. The decision threshold is the probability (from 0 to 1) of the model to classify a case as a positive or a negative. With a threshold set at 0.5, the model classifies cases with probabilities above this value as positive, and those below as negative. Where area under the ROC curve (AUC) is 0.5, the AI performance is similar to a random classifier.

Let's consider a screening model that utilizes binary classification:

Positive cases: people who have the disease;
Negative cases: people who do not have the disease.

The AI model generates the probability scores for target disease presence. To classify cases into “pathology” or “normal,” we can set different decision thresholds. For instance, at a threshold of 0.7 on ROC curve, the model classify cases as “pathology” when the disease probability exceeds 70%. Lowering the threshold to 0.3 increases the model sensitivity for disease detection but may increase false-positive classifications of healthy individuals.

**Figure 1:**
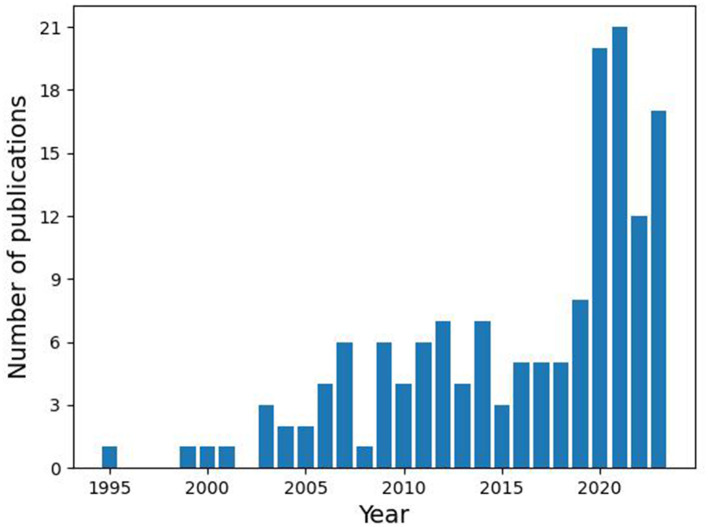
ROC curve and its components. (x, y) – maximum value of the Youden index for the ROC curve, d – the shortest distance from the point (0, 1) to the ROC curve, J – Youden index identification, NB – the net benefit decision threshold.

Currently, there are several main ways to determine the most appropriate decision threshold in diagnostic tests with continuous outcomes:

1. Assigning equal weights to sensitivity and specificity (maximizing both AI performance metrics). This represents the point on the ROC curve where the distance (d) from coordinates (0,1) to the curve reaches its minimum (Figure 1).
2. The second method for optimizing sensitivity and specificity of the AI model and determining the optimal decision threshold is the Youden index (Martínez-Camblor and Pardo-Fernández, 2019). The Youden index is the distance from the line of equality to the ROC curve (Figure 1) calculated using the following formula: J = sensitivity + specificity – 1 (Youden, 1950).
3. The third method: manually assigning predetermined sensitivity or specificity values. In autonomous AI MMG screening, setting the threshold to 100% sensitivity minimizes the risk of missed pathologies. In this scenario, the radiologist would only analyze studies with suspected pathological findings (Vasilev et al., 2024).
4. The fourth method: determining a decision threshold using a net benefit parameter (Pfohl et al., 2022). The latter may factor in the following components: financial costs of false-positive (FP) and false-negative (FN) diagnoses, patient's comfort (as a result of positive diagnosis treatment is required), costs associated with additional examinations, and other expenditures. The final method for calculating net benefit depends on which features researchers choose to include in their study. For this reason, net benefit analysis is rarely applied in clinical trials due to its computational complexity and challenges in interpretation (Greiner et al., 1995; Kumar and Indrayan, 2011).

The first two approaches to determining a decision threshold utilize graphical ROC analysis seeking the point with the highest sensitivity-to-specificity ratio closest to coordinates (0,1) (Figure 1). In contrast, in the net benefit analysis precedes the graphical search.

**Figure 2:**
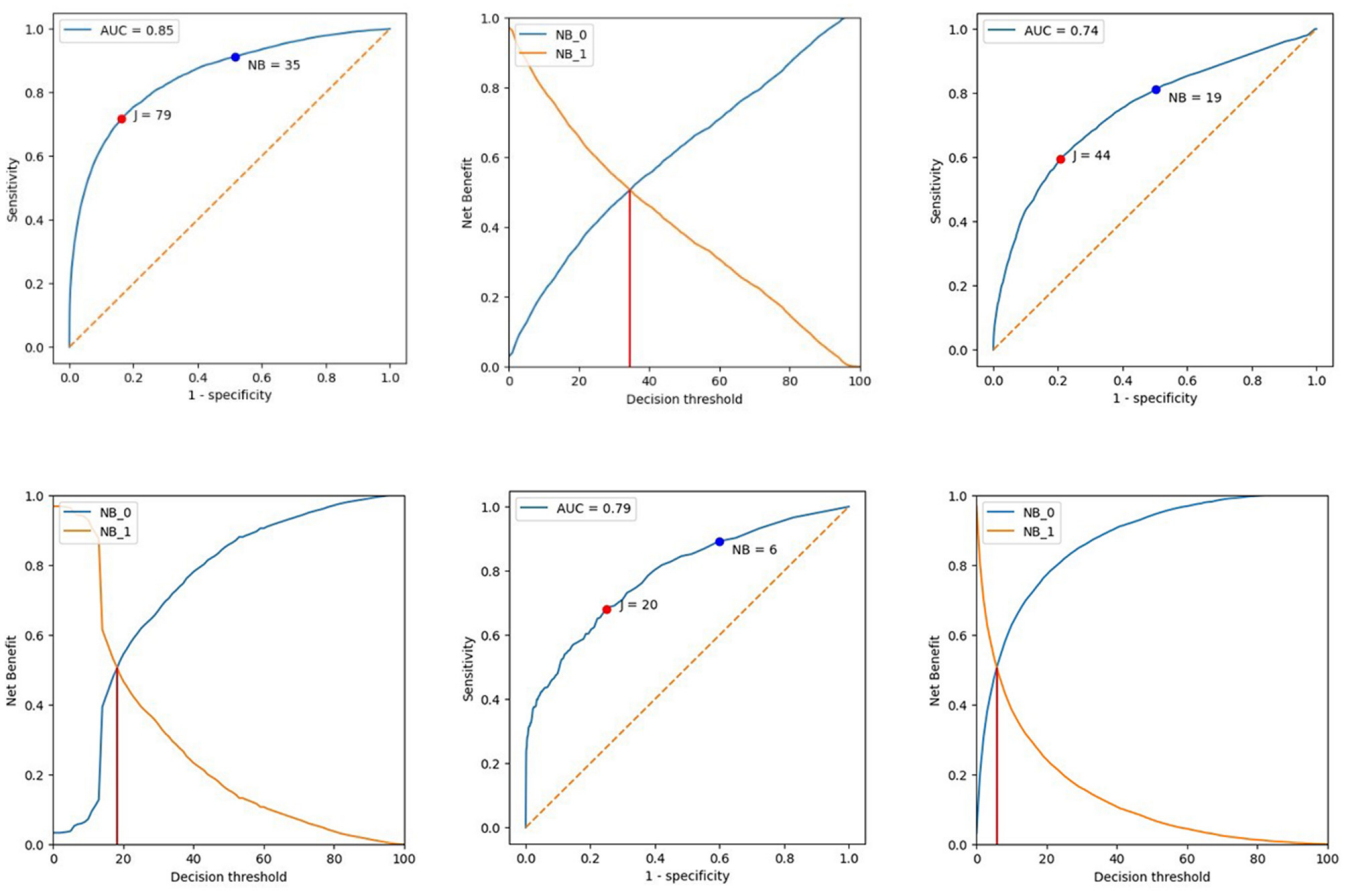
PubMed publications containing the “Net benefit” term in the title.

Net benefit is a quantifiable metric that has recently seen growing adoption among healthcare and AI researchers (Pencina et al., 2008; Vickers et al., 2016). Historically, the derivation of the net benefit Formula was first published by Peirce in 1884 (Peirce, 1884). Introducing such a formula meant to measure the predictive performance across diverse domains including medicine, economics, meteorology and other areas where prediction accuracy is crucial. Since 2019, the PubMed database has reported growing adoption of the “net benefit” term in healthcare publications (see Figure 2). The literature suggests utilizing a costs/benefit analysis to calculate the net benefit parameter that would help determine a decision threshold from the clinical utility perspective both for doctors and for patients (Singh et al., 2023).

Vickers, Van Calster, and Steyerberg provide a detailed example of applying the net benefit parameter in the clinical practice (Vickers et al., 2016), without the use of AI. The paper examines the application of the net benefit in deciding whether to perform a prostate biopsy based on elevated prostate-specific antigen (PSA) levels. This procedure is associated with additional burden (L – the Losses) for healthcare medical facilities such as surgical and histological costs, as well as patient discomfort and biopsy-related risks. The benefit (P - Profits), i.e., the prevented disease, is calculated from all of these factors. The net benefit parameter is consistently applied across most publications on this topic. Fundamentally, the calculations of clinical or economic benefits (profits and losses) for both healthcare providers and patients follow the similar approach to the PSA case (Vickers et al., 2019; Pfohl et al., 2022; Ehrmann et al., 2023). The paper “Calculating and Interpreting ICERs and Net Benefit” (Paulden, 2020), introduces two further approaches for calculating the net benefit in healthcare systems: “net health benefit” and “net monetary benefit.” In summary, the fundamental principle is to treat an individual *i* only if the probability p*i* of individual *i* having the disease exceeds the expected loss (1-p*i*)^*^L. This means that anticipated benefits outweigh the potential drawbacks (i.e., p*i* probability (P) > (1-p*i*)^*^L) (Rousson and Zumbrunn, 2011).

Thus, (1-pi)^*^L can used to calculate mathematical expectation of risks and the treatment costs. The mathematical expectation represents the weighted average of possible outcomes, where each outcome is multiplied by its probability and then summed. In this context, (1-pi)^*^L is one of the possible outcomes at a given probability. As previously noted, L represents the cost or loss (e.g., mental stress) associated with an undesirable outcome (such as an unnecessary prostatectomy) and (1-pi) is the probability of such outcome occurring. In both scenarios, these calculation parameters are not applicable to AI. However, in the above-mentioned publication, the author presents an approach to estimating the net benefit for AI, suggesting that a surgery can be considered when the expected benefit exceeds the loss. This requires calculating various decision thresholds (probabilities) (Formula 1):


$$\begin{array}{l} \text{pt }=\text{ L}/\left(\text{L}+\text{P}\right) \end{array}$$ (1)

where P – benefits (profits) from treatment of sick patients, L – losses from treatment of healthy individuals. In Formula 1, the probability threshold pt is defined as pt/(1 – pt) = L/P. In other words, this represents how clinicians or patients judge the relative harm from false positive and false negative results (Rousson and Zumbrunn, 2011).

Using probability thresholds, a graphical representation can be constructed with the net benefit on the y-axis and decision thresholds on the x-axis (Rousson and Zumbrunn, 2011). This allows a researcher to visually evaluate the net benefit model and the selected decision thresholds. The resulting probability is then used in the net benefit Formula for AI.

This approach is reasonable, given the strong dependence of the country's economy on the timely disease diagnosis. For instance, in the United States, breast cancer mortality (accounting for one-fifth of all deaths among women over 50) resulted in $84.7 billion in indirect economic losses in 2000 (Radice and Redaelli, 2003). Further research has shown that these economic losses persisted over time. By 2015, breast cancer mortality had cost the United States $8.1 billion (or 0.24% of the annual Gross Domestic Product). By 2030, this figure will likely remain significantly high ($7.5 billion USD, 0.14% of the Gross Domestic Product) (Barchuk et al., 2019). A similar situation is observed in Russia, China, the UK and other countries. The early diagnosis and timely treatment of breast cancer are important components of the country's economic wellbeing (Pashayan et al., 2018; Wang et al., 2022). The interpretation of the net benefit parameter in clinical tasks and its application can vary.

This study aims to develop a methodology for determining a decision threshold on the ROC curve through net benefit computation, with the goal of enhancing the utility of AI applications in patient care by integrating quality metrics to maximize patient benefit.

In this section, we have highlighted the unique contributions of our study to the existing body of knowledge. This study introduces a novel approach to determining the decision threshold on the ROC curve using net benefit parameter. This method combines traditional quality metrics in a unique way, enhancing the applicability and effectiveness of AI in healthcare settings. Additionally, we outline the practical implications of our findings, proposing actionable strategies for the implementing our methodology in clinical setting and applying AI to MMG screening.

## Methods

This is a retrospective observational cohort study.

Ethical expertise: The data were obtained from the Experiment on the use of innovative technologies in the field of computer vision for the analysis of medical images and further use in the healthcare system of Moscow [https://mosmed.ai/en/]. This study was registered in ClinicalTrials (NCT04489992).

This study analyzed data from three AI services participating in the Experiment.

The dataset comprised 663,606 unsampled digital mammograms meeting the inclusion criteria, acquired in Moscow public medical facilities between July 22, 2020 and December 29, 2022. The data were downloaded from the Unified Radiological Information Service of the Unified Medical Information and Analytical System (URIS UMIAS) of Moscow, the Russian Federation (RF) and de-identified by removing tags containing personal data using a dedicated software developed by our organization [http://dicom.nema.org/Medical/dicom/2016d/output/chtml/part03/sect_C.2.2.html].

Inclusion criteria were female sex and age over 40 years. This age criterion aligns with the Russian clinical guidelines for breast cancer screening initiation at age 40 (Pashayan et al., 2018). This corresponds to the recommendations of the World Health Organization (2022). Women who underwent mammography at an earlier age likely had some abnormalities, so including them in the sample would be unreasonable. Another criterion was literature data indicating increased risk of breast cancer after 35 years old, affecting approximately one in eighth women (12% of the female population) (Kim et al., 2023). The age distribution in the final dataset was as follows: min 40, max 98, median 59.

The other inclusion criteria were the availability of AI service's results and a radiology report. The exclusion criterion was the absence of BI-RADS classification in the report. The imaging studies were verified by analyzing text reports using a natural language processing algorithm. While detailed algorithm description is beyond the scope of this paper, more details can be found in Morozov et al. (2020a,b). The BI-RADS scores assigned by radiologists were converted to binary classification: BI-RADS 4-6 were labeled as “pathology” (1), BI-RADS 1-3 were labeled as “normal” (0). BI-RADS category 0 was excluded as it indicates an error in mammographic evaluation. Also, since BI-RADS 3 represents less than 2% chance of malignancy% it was classified as “normal” (Berg et al., 2020). For each patient in the dataset we added the binary column “Presence or absence of pathology,” where 1 indicated “pathology” and 0 indicated “normal.” The class distribution in the final dataset was as follows: 644,165 patients (97.1%) were classified as “normal,” while 19,441 patients (2.9%) were classified as having “pathology.”

### Artificial intelligence services

The digital MMGs were processed by three AI services that generated a pathology probability for each study (Dembrower et al., 2020; Kim et al., 2020; Salim et al., 2020; Eghtedari et al., 2021; Pavlovich et al., 2021). For more information about the AI services, please see the References section. Be advised that their architecture and core algorithms are trade secrets. During the study, two models (AI #1, AI #2) experienced three version changes, while one model (AI #3) had no version changes.

### Statistical analysis

Sample size for modeling: a preliminary sample size was not calculated. The dataset includes all the MMGs processed by the AI services acquired within the period of interest that match the inclusion criteria. The MMGs were distributed between AI services (Table 1).

**Table 1:** The final sample (number of patients).

**Service version**	**Name of the AI service**		
	**AI #1**	**AI #2**	**AI #3**
1	90949	4922	9481
2	212968	43630	
3	241445	60211	

Statistical analysis: The data analysis was carried out using Anaconda distribution of Python programming language, along with pandas, NumPy, matplotlib, and scikit-learn libraries distributed under the GNU General Public License. All computations and data visualization were performed in Jupyter Notebook. The net benefit calculation was performed for each version of each service. As previously discussed, the standard formula for net benefit considers ratio of true-positives to the total number of measurements, adjusted by proportion of false-positives multiplied by the probability ratio of disease presence vs. absence. The AI performance was assessed based on its ability to correctly identify pathology and its propensity for false alarms.

### Net benefit

The literature describes the use of the net benefit parameter to evaluate adjustments to the AI model parameters in the context of hospital operations or in response to varying clinical scenarios (Vasilev et al., 2023b). The original formula for net benefit (Formula 2), proposed by Vickers et al. (2016), considers both the doctor's and patient's perspectives, depending on the AI's decision about the presence of an abnormality. Formula 2 provides a logical link to the probability threshold (pt) from Formula 1, enabling the classification of cases as “normal' vs. “pathology.” This classification accounts for both true-positive and false-positive outcomes. The formula is as follows:


$$\begin{array}{l} \text{Net Benefit}=\;\frac{\text{TruePositiveCount}}{n}\;-\;\frac{\text{FalsePositiveCount}}{n}\\ \;\times \;\left(\frac{p_{t}}{1-p_{t}}\right), \end{array}$$ (2)

where pt – decision threshold probability on the ROC curve that distinguishes the sick (true positives) from healthy individuals with positive test results (false positives); n – the total number of measurements. Understanding and applying this formula has the potential to significantly impact the healthcare industry by improving diagnostic accuracy and patient outcomes.

This study explores the potential application of the net benefit concept to adjust the decision thresholds within AI systems, with the ultimate goal of optimizing patient routing. Specifically, the aim is to ensure prompt referral of women suspected of having breast malignancies to dedicated medical professionals. We suggest using the net benefit parameter to calculate the optimal decision threshold on the ROC curve, a novel approach not previously employed. Unlike conventional metrics that evaluate sensitivity and specificity independently, our net benefit parameter consolidates these aspects into a unified metric. This approach secures a more comprehensive assessment of clinical AI performance and distinguish it from traditional clinical metrics.

We modified Formula 2 to consider only true-positive (TP) cases (women with breast cancer) and true-negative (TN) cases (women without breast cancer), excluding false-positive interpretations. We propose two expressions for calculating net benefit. The first modification enhances the standard formula to better reflect the AI's ability to distinguish between the presence or absence of pathology. As a result, we obtained the Net Benefit0 formula (Formula 3):


$$\begin{array}{l} {\text{Net Benefit}}_{0}=\;\frac{\text{TruePositiveCount}}{n}\;-\;\frac{\text{TrueNegativeCount}}{n}\\ \;\times \;\left(\frac{p_{0}}{1-p_{0}}\right). \end{array}$$ (3)

Next, we recalculated the metric to include only false-negative (FN) (breast cancer undetected by the AI) and false-positive (FP) (incorrectly detected breast cancer) cases. Thus, the second modification focused on diagnostic errors most critical for patients and healthcare systems, which yielded the Net Benefit1 formula (Formula 4):


$$\begin{array}{l} {\text{Net Benefit}}_{1}=\;\frac{\text{FalsePositiveCount}}{n}\;-\;\frac{\text{FalseNegativeCount}}{n}\\ \;\times \;\left(\frac{p_{1}}{1-p_{1}}\right) \end{array}$$ (4)

where p0 – a probability of the ‘normal' outcome calculated using Formula 1; p1 – a probability of the ‘pathology' outcome calculated using Formula 1; n – total number of measurements.

**Figure 3:**
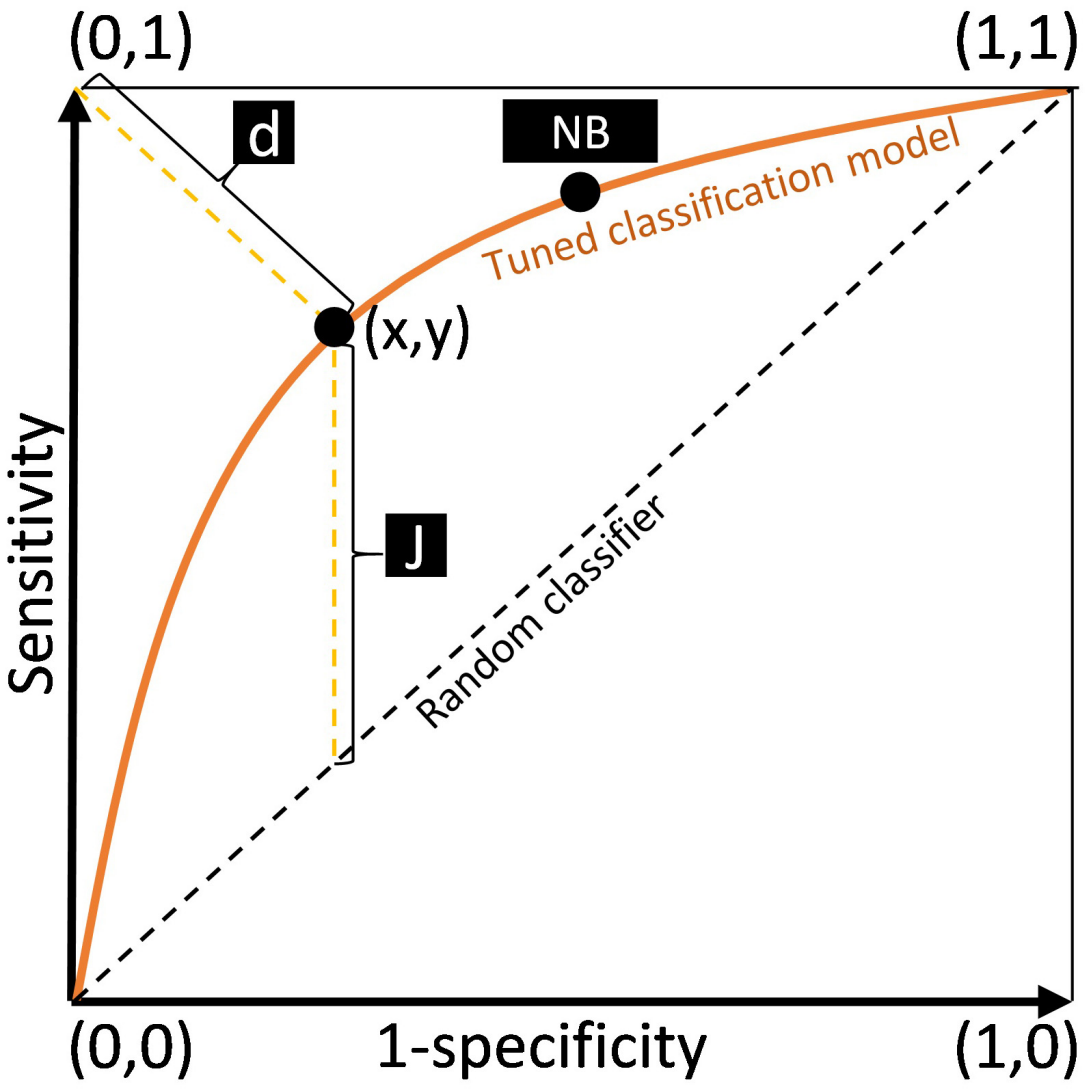
ROC curves and decision threshold. 1 – ROC curve showing decision threshold derived from Youden index (J) and NetBenefit0 and NetBenefit1 intersection (NB). 2 – Net benefit decision threshold determined by NetBenefit0 (NB_0) and NetBenefit1 (NB_1) curve intersections. A – AI 1 (version: 3); B – AI 2 (version: 3); C – AI 3.

Using Formulas 3, 4, we plotted a chart containing NetBenefit0 and NetBenefit1 curves for the probability values from the dataset. Next, we determined a decision threshold by the x-coordinate of the intersection point between NetBenefit0 and NetBenefit1 curves. The intersection is the optimal threshold that represents the maximum overall benefit (net benefit). This threshold represents the balance between identifying true-positive cases and minimizing false-positives and false-negatives. By achieving this balance, the diagnostic utility is optimized, considering all potential outcomes. Specifically, it identifies a threshold where the aggregated benefit from accurate diagnoses outweighs the risks associated with false and missed cases. This approach optimizes diagnostic efficacy and maximizes patient and health outcomes. An example of a chart and a decision threshold calculation is shown in Figure 3(2).

The net benefit parameter was used to optimize the classification model by determining the optimal probability threshold, given the costs and losses associated with misclassification (equal number of “normal” and “pathology” classes).

Formulas 3, 4 maximize the net benefit value (since the intersection represents equality of TP and FP) and minimize the number of false-negative (FN) observations.

We constructed a confusion matrix for all versions of the AI models using the decision thresholds derived from the net benefit parameter and the Youden index. Next, we compared the results against radiologist-verified ground truth.

Confusion matrix – a fundamental statistical tool for summarizing the performance of classification algorithm in machine learning. It generates a spreadsheet of actual vs. predicted classifications for a comprehensive evaluation of the model's accuracy. The matrix consists of four primary components: true-positives, true-negatives, false-positives, and false-negatives. These components are used for calculating various metrics, including precision, recall, specificity, and the F1 score. By analyzing these metrics, researchers reveal the strengths and weaknesses of their classification models, facilitating informed decisions on model selection and optimization strategies.

Comparisons were made using McNemar's test. The absence of statistically significant differences between the services' results and the reference was taken as the null hypothesis. An alternative statistical hypothesis was the presence of statistically significant differences with a *p*-value < 0.05.

The McNemar test is a non-parametric statistical method designed for analyzing paired nominal data. It assesses whether changes observed between paired samples are statistically significant.

To evaluate the effect of decision threshold changes on the final AI outcomes, we calculated the diagnostic accuracy metrics [i.e., specificity (Sp), sensitivity (Se), and accuracy (Acc)] for the decision thresholds using the net benefit parameter and the Youden index.

Sensitivity: The ability of a diagnostic test to correctly identify sick individuals, calculated as the proportion of true-positives in the diagnosed population (Formula 5):


$$\begin{array}{l} \text{Sen }=\;\frac{\text{TP}}{\text{TP }+\text{ FN}}. \end{array}$$ (5)

Specificity: The ability of a diagnostic test to correctly identify individuals who do not have a condition, calculated as the proportion of true-negatives in the diagnosed population (Formula 6):


$$\begin{array}{l} \text{Sen }=\;\frac{\text{TN}}{\text{TN }+\text{ FP}}. \end{array}$$ (6)

Accuracy: The share of true results (both true positives and true negatives) among all cases, measuring the overall correctness of a diagnostic test (Formula 7):


$$\begin{array}{l} \text{Acc }=\;\frac{\text{TP }+\text{ TN}}{\text{TP }+\text{ TN }+\text{ FP }+\text{ FN}}. \end{array}$$ (7)

**Figure 4:**
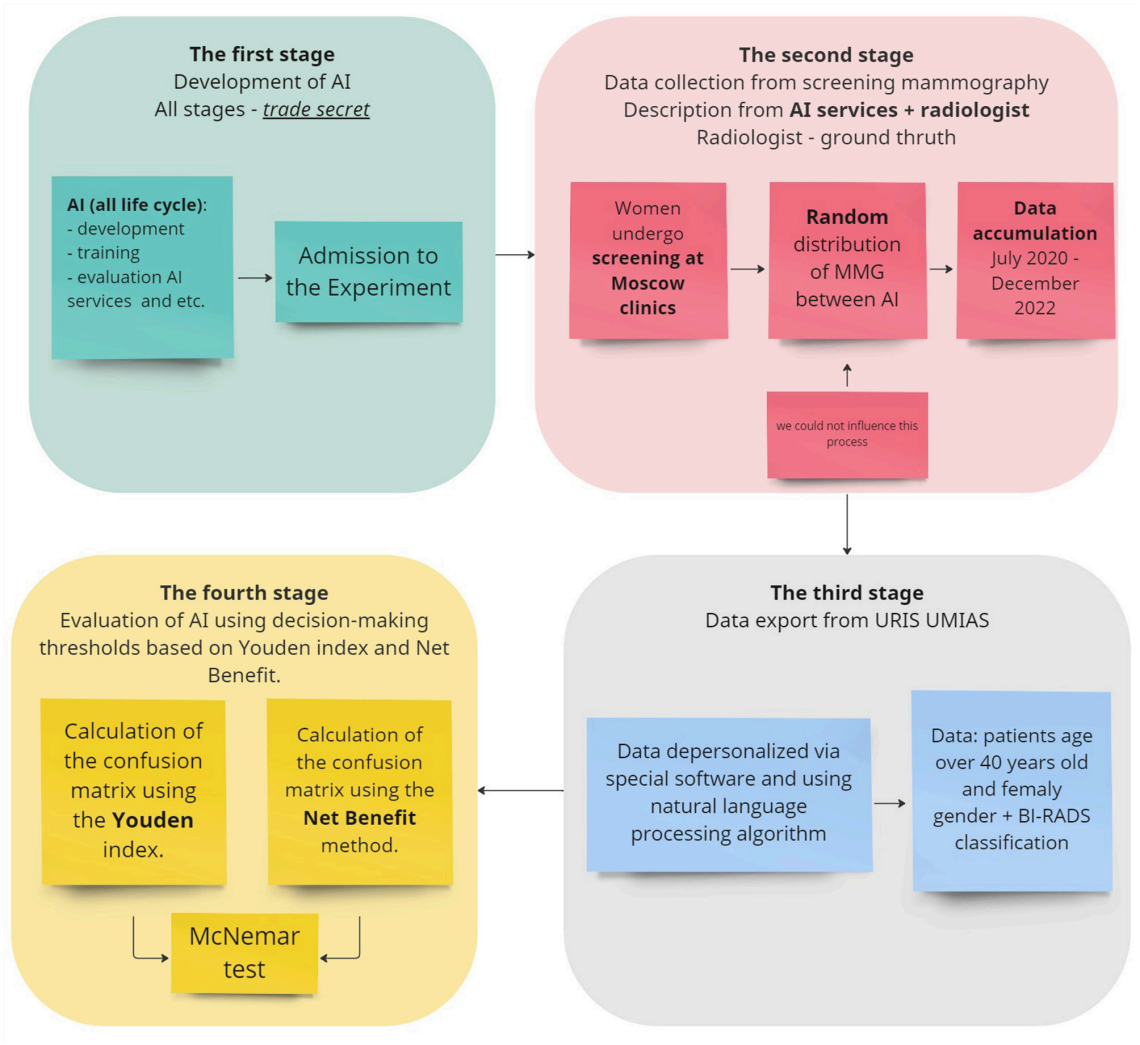
The study design.

For a clearer understanding, the study design is illustrated in the Figure 4.

## Results

To evaluate the results, we built ROC curves with two points representing the decision thresholds calculated using the Youden index and the net benefit parameter [Figure 3(1)]. Their exact values and diagnostic accuracy metrics are presented in Tables 2, 3 for each version of each AI service.

**Table 2:** AI diagnostic metrics using Youden index (J) threshold values.

**Artificial Intelligence service and version**							
**Criterion**	**AI #1**			**AI #2**			**AI #3**
	**Ver_1**	**Ver_2**	**Ver_3**	**Ver_1**	**Ver_2**	**Ver_3**	**1**
Threshold-J (probability)	0.57	0.78	0.79	0.44	0.42	0.44	0.20
Se^*^	0.53	0.68	0.72	0.58	0.66	0.60	0.68
Sp^*^	0.84	0.81	0.84	0.84	0.76	0.79	0.75
Acc^*^	0.83	0.81	0.84	0.83	0.75	0.78	0.75

^*^Se – sensitivity, Sp – specificity, Acc – accuracy.

**Table 3:** AI diagnostic metrics using net benefit (NB) threshold values.

**Artificial Intelligence service and version**							
**Criterion**	**AI #1**			**AI #2**			**AI #3**
	**Ver_1**	**Ver_2**	**Ver_3**	**Ver_1**	**Ver_2**	**Ver_3**	**1**
Threshold-NB (probability)	0.17	0.51	0.35	0.21	0.21	0.19	0.6
Se^*^	0.80	0.88	0.91	0.85	0.84	0.99	0.85
Sp^*^	0.49	0.49	0.48	0.51	0.50	0.50	0.49
Acc^*^	0.50	0.50	0.50	0.52	0.51	0.51	0.50

^*^Se – sensitivity, Sp – specificity, Acc – accuracy.

As shown in Table 2, specificity, sensitivity, and accuracy vary with the Youden index. For instance, AI #1 version 3 achieved the highest accuracy (Youden index = 0.79), with both sensitivity and specificity reaching optimal values.

For AI #1 version 3, the optimal threshold probability was 0.35. At this threshold, the algorithm achieved the highest sensitivity, while maintaining similar levels of specificity and accuracy.

Table 4 presents McNemar test *p*-values comparing the radiologist conclusion (Ground Truth) against AI predictions using both Youden index (J) and net benefit (NB) thresholds.

**Table 4:** McNemar test *p*-values applied to the confusion matrix comparing the radiologist conclusion (Ground Truth) against AI predictions using both Youden index (J) and net benefit (NB) thresholds.

**Artificial Intelligence service and version**, ***p*****-value**							
**Matrix by criterion**	**AI #1**			**AI #2**			**AI #3**
	**Ver_1**	**Ver_2**	**Ver_3**	**Ver_1**	**Ver_2**	**Ver_3**	**1**
**Decision-making threshold by Youden Index (J)**							
GT	< 0.0001	< 0.0001	< 0.0001	< 0.0001	< 0.0001	< 0.0001	< 0.0001
Se^*^	< 0.0001	< 0.0001	< 0.0001	< 0.0001	< 0.0001	< 0.0001	< 0.0001
Sp^*^	< 0.0001	< 0.0001	< 0.0001	< 0.0001	< 0.0001	< 0.0001	< 0.0001
**Decision-making threshold by Net Benefit (NB)**							
GT	< 0.0001	< 0.0001	< 0.0001	< 0.0001	< 0.0001	< 0.0001	< 0.0001
Se^*^	< 0.0001	< 0.0001	< 0.0001	< 0.0001	< 0.0001	< 0.0001	< 0.0001
Sp^*^	< 0.0001	< 0.0001	< 0.0001	< 0.0001	< 0.0001	< 0.0001	< 0.0001

^*^Se – sensitivity, Sp – specificity.

The AI results differed significantly from the radiologist interpretations, regardless of the threshold determination method, while still exhibiting classification errors.

Table 5 presents McNemar test *p*-values. The normal/pathology classes assigned using the Youden index were regarded as the reference. The AI results based on the net benefit decision thresholds were regarded as an alternative opinion.

**Table 5:** McNemar test *p*-values: Comparison of the net benefit (NB) confusion matrix vs. the Youden index (J) confusion matrix as Ground Truth (GT).

**Parameter**	**AI #1**			**AI #2**			**AI #3**
	**Ver_1**	**Ver_2**	**Ver_3**	**Ver_1**	**Ver_2**	**Ver_3**	**1**
NB threshold	0.17	0.51	0.35	0.21	0.21	0.19	0.6
*p*-value	< 0.0001	< 0.0001	< 0.0001	< 0.0001	< 0.0001	< 0.0001	< 0.0001

The McNemar test revealed significant differences in AI classifications between Youden index and net benefit threshold methods.

Tables 1, 2 demonstrate that our decision threshold method yielded an increase in mean sensitivity from 64% to 87% and a decrease in mean specificity from 80% to 49% across all versions of the three AI services. Possible clinical consequences outweigh the risk of missing breast cancer, which would be more costly than retesting after a false-positive result. Using the Russian universal healthcare rates as an example, we compared the economic burden from a false-positive algorithm outcome to that of a late diagnosis scenario. Screening: The cost of a single mammography acquisition averages 464.96 rubles (US$5.75, at the October 2025 exchange rate). The total cost of breast cancer screening amounts to 2492.5 rubles (US$30.83). The cost of a follow-up visit for suspicious abnormalities detected during a regular checkup is 329 rubles (US$4.07). In addition, the cost of follow-up diagnostic procedures (unavoidable if suspicious abnormalities are detected) per patient visit is 1,599.8 rubles (US$19.79). Therefore, the maximum cost incurred by a false-positive algorithmic outcome is 4,886.26 rubles (US$60.43) (if the diagnosis is not confirmed). The cost of breast cancer treatment varies across the disease stages: stage 1 costs 305,131 rubles per patient (US$3,773.81), stage 2 costs 425,564 rubles (US$5,263.31), stage 3 costs 553,038 rubles (US$6,839.89), and stage 4 costs 545,303 rubles (US$6,744.23), not considering the socioeconomic impact on the family in the event of patient's death. These data are based on breast cancer diagnosis costs in the Russian Federation published in 2024 (Ignatyeva et al., 2024).

## Discussion

Digital mammography for early breast cancer screening is being widely implemented worldwide (Morozov et al., 2019). Timely diagnosis and treatment improve patient quality of life and reduce breast cancer-related mortality (Arzamasov et al., 2023). Early breast cancer detection is crucial for patient outcomes, national economies and healthcare systems. Nevertheless, specialists miss up to 31% of breast cancers on mammograms, which is why this method involves double reading (Seely and Alhassan, 2018; Taylor-Phillips and Stinton, 2020). Specialist shortages increase radiologist workload and report preparation time (Le et al., 2019; Kudryavtsev et al., 2024). These challenges can be addressed by introducing AI-powered software into clinical practice and expanding the respective regulatory framework (Ovsyannikov et al., 2020).

Our previous paper proposed national criteria for classifying AI as a medical device based on the ROC curve analysis, and outlined strategies for cancer screening (Arzamasov et al., 2023). For clinical practice approval, AI developers must provide metrics whose values depend on the decision threshold for the normal/pathology classes. Meta-analyses compared sensitivity and specificity of double reading by radiologists and AI in mammography (Hickman et al., 2022; Liu et al., 2022). The studies established diagnostic accuracy criteria (Arzamasov et al., 2023), showing that radiologist's sensitivity reaches 67% at the lower limit of the confidence interval, with specificity ranging from 87 to 100%. Sensitivity and specificity of AI models were determined based on the Youden index threshold and found to be comparable to the radiologists'. Nevertheless, the best AI model demonstrated the lower limit of the sensitivity confidence interval of 73%, meaning up to 27% of pathologies remain undetected. Nevertheless, in breast cancer screening, and given the financial burden presented in the “Results” section, detection remains the highest priority. While setting the decision threshold using Youden index yields the highest accuracy of the machine learning algorithms by establishing the optimal balance between specificity and sensitivity, this approach is not what is required for screening. For this scenario, the NB approach proved to be the most cost-effective. Although the algorithmic accuracy decreases, the risk of missing breast cancer is minimized, which is more important given the potential corresponding clinical outcomes and mortality rate. At the same time, as algorithms evolve and improve their accuracy (Vasilev et al., 2025), we anticipate that the rate false positives will decrease, making our method even more relevant in the future.

This study evaluates the net benefit parameter as a novel approach for determining the decision threshold for the “normal” vs. “pathology” classes. It was found that this method triples the number of FP observations and doubles TP by AI. In addition, FN decreased by half in comparison with the Youden index thresholds. This methodology represents a compromise between maximizing the accuracy of positive findings and minimizing false outcomes. The focus on increasing TP at the expense of reducing FN leads to an increase in FP. This occurs because a strategy that prioritizes positive classes would increase the risk of misclassifying negative cases. Tables 2, 3 show that both accuracy and specificity of the AI services are higher where the Youden index threshold is used. In contrast, sensitivity is always higher with the net benefit decision thresholds. It should be specified that sensitivity indicates the model's ability to identify true-positive cases (TP) (a pathology presence) among TP and FN.

The FP rate from the net benefit threshold is almost four times higher than for Youden index. This suggests an overcautious AI producing more false-positive observations. When calculating the net benefit decision threshold, the goal was to make AI emulate an overcautious radiologist.

In mammographic cancer detection screening, sensitivity is critical for the model applicability. A model that better identifies TP is more beneficial for patient outcomes, since a disease will not be missed and medical care will be provided on time. Early cancer detection contributes to better treatment effectiveness, reduces mortality and the healthcare burden (Geras et al., 2019).

McNemar test analysis of AI results using both Youden index and net benefit thresholds (Table 4) showed statistically significant differences (*p*-value < 0.05) from radiologist reference values. Similar differences were observed between the AI results obtained with the Youden index as a reference vs. the metrics obtained with net benefit thresholds (see Table 5). Statistically significant differences in the McNemar test results indicate systematic variations between AI and radiologist classification strategies. This discrepancy could be attributed to variations in priorities or prioritization of different critical errors depending on the task. Given the large sample sizes in our study (ranging from 4,922 to 241,445 cases in datasets), the McNemar test achieved very high statistical power. Consequently, even modest differences in classification patterns between the two threshold methods and radiologists resulted in highly significant *p*-values (all *p* < 0.0001). To ensure appropriate interpretation, we report both statistical significance (Tables 4, 5) and effect sizes (absolute differences in performance metrics, Tables 2, 3) to distinguish between statistical and clinical significance.

The study showed that the net benefit decision threshold is more beneficial when AI is used in medical screening, where spotting a life-threatening condition is critical. In addition to improved TP rate, models using net benefit thresholds show lower FN rate, reducing missed diagnoses. This approach can be used to improve classification by optimizing the balance between different types of errors. However, an increased FP rate can have significant drawbacks and undesirable consequences. Carefully weighing the pros and cons when choosing a classification strategy is crucial. Since there is statistically significant difference between the AI and the radiologist interpretations, combined with the maximum net benefit and radiologist's high specificity (Arzamasov et al., 2023), double reading is still recommended for digital MMG. Here, the initial reading may be handled by AI, whereas the second reading must be carried out by a radiologist to maximize both sensitivity and specificity. Therefore, the double reading approach is the most rational. It minimizes costs because only one radiologist interprets an MMG while the AI replaces the second specialist. Unlike the maximum sensitivity setting, our method secures the radiologist's trust in AI results. Moreover, maximizing sensitivity substantially increases false-positive rates which could lead radiologists to disregard AI results entirely. This would negatively affect both the quality of diagnosis and the integration of AI into healthcare.

This paper offers a new perspective on determining decision thresholds for AI services in digital MMG. It also emphasizes the importance of further application of the net benefit parameter to AI services in breast cancer screening (Arzamasov et al., 2023). We suggest that the net benefit approach will facilitate integration of AI in mammography screening workflows. When used as a primary reader alongside a human radiologist, AI significantly enhances diagnostic accuracy in mammography. This method minimizes the costs associated with unnecessary consultations, and capitalizes on the unique strengths of both AI and human expertise. This combination secures comprehensive evaluation, allowing for a nuanced understanding and handling of complex cases, ultimately improving patient care and outcomes.

Despite its advantages, this methodology has limitations, including model bias (overcautiousness) evidenced by McNemar test differences from radiologist reference opinions, and increased false-positive rates. These results are specifically optimized for mammography screening applications. It is important to keep in mind that selection of the optimal decision threshold requires additional research, depending on the task. Further validation of this methodology using datasets from other medical domains and populations is essential.

Another limitation of our study is that the very large sample sizes, while providing robust statistical estimates, resulted in all McNemar tests yielding *p* < 0.0001, making it difficult to distinguish the relative importance of differences across service versions based on *p*-values alone. We addressed this by reporting effect sizes and focusing on clinical interpretation of the magnitude of differences.

Summary of the main study result:

The net benefit parameter is applicable to configure AI models used in various fields of radiology (such as mammography). This approach enables effective binary classification between “normal” (0) vs. “pathology” classes (1). Unlike the Youden Index, the net benefit parameter will increase the number of correctly identified diseases while reducing false-negative results.

## Conclusions

This study demonstrated that configuring AI services with the net benefit parameter significantly improves sensitivity compared to Youden index, thereby reducing the likelihood of missed breast cancer.

The proposed method for determining decision thresholds for AI services can be beneficial in the domains beyond breast cancer screening. Such configuration can be applied in medical genetics for detecting rare gene patterns that might be otherwise overlooked by humans. In psychiatry, AI classification can quickly and accurately identify patients urgently needing help. In neurological applications, such as diagnosing neurodegenerative diseases or acute conditions, AI may require high sensitivity and specificity despite the boost in false-positives. Thus, the proposed method can be used in medicine and many other domains that rely on high accuracy and rapid diagnosis establishment.

## Data Availability

The datasets presented in this article are not readily available because it is owned by the Moscow Health Department. Requests to access the datasets should be directed to npcmr@zdrav.mos.ru or https://mosmed.ai/datasets/.

## References

[B1] Arzamasov K. M. Vasilev Yu A. Vladzymyrskyy A.V. Omelyanskaya O. V. Bobrovskaya T. M. Semenov S. S. . (2023). The use of computer vision for the mammography preventive research. Profilakticheskaya meditsina 26:117. doi: 10.17116/profmed202326061117

[B2] Bae M. S. Moon W. K. Chang J. M. Koo H. R. Kim W. H. Cho N. . (2014). Breast cancer detected with screening US: Reasons for nondetection at mammography. Radiology 270, 369–377. doi: 10.1148/radiol.13130724 24471386

[B3] Barchuk A. Bespalov A. Huhtala H. Chimed T. Belyaev A. Moore M. . (2019). Productivity losses associated with premature mortality due to cancer in Russia: a population-wide study covering 2001–2030. Scand. J. Public Health 47, 482–491. doi: 10.1177/1403494819845565 31313982 PMC6651608

[B4] Berg W. A. Berg J. M. Sickles E. A. Burnside E. S. Zuley M. L. Rosenberg R. D. . (2020). Cancer yield and patterns of follow-up for BI-RADS category 3 after screening mammography recall in the national mammography database. Radiology 296, 32–41. doi: 10.1148/radiol.2020192641 32427557

[B5] Cho S. Kim Y. J. Lee M. Woo J. H. Lee H. J. (2021). Cut-off points between pain intensities of the postoperative pain using receiver operating characteristic (ROC) curves. BMC Anesthesiol. 21, 1–8. doi: 10.1186/s12871-021-01245-5 33494704 PMC7831264

[B6] Dembrower K. Wåhlin E. Liu Y. Salim M. Smith K. Lindholm P. . (2020). Effect of artificial intelligence-based triaging of breast cancer screening mammograms on cancer detection and radiologist workload: a retrospective simulation study. Lancet Digital Health 2, e468–e474. doi: 10.1016/S2589-7500(20)30185-0 33328114

[B7] Eghtedari M. Chong A. Rakow-Penner R. Ojeda-Fournier H. (2021). Current status and future of BI-RADS in multimodality imaging, from the AJR special series on radiology reporting and data systems. Am. J. Roentgenol. 216, 860–873. doi: 10.2214/AJR.20.24894 33295802

[B8] Ehrmann D. E. Joshi S. Goodfellow S. D. Mazwi M. L. Eytan D. (2023). Making machine learning matter to clinicians: model actionability in medical decision-making. *npj Digit. Med*. 6, 1–5. doi: 10.1038/s41746-023-00753-7 36690689 PMC9871014

[B9] Evans K. K. Birdwell R. L. Wolfe J. M. (2013). If you don't find it often, you often don't find it: why some cancers are missed in breast cancer screening. PLoS ONE 8, 1–6. doi: 10.1371/journal.pone.0064366 23737980 PMC3667799

[B10] Geras K. J. Mann R. M. Moy L. (2019). Artificial intelligence for mammography and digital breast tomosynthesis: Current concepts and future perspectives. Radiology 293, 246–259. doi: 10.1148/radiol.2019182627 31549948 PMC6822772

[B11] Greiner M. Sohr D. Göbel P. (1995). A modified ROC analysis for the selection of cut-off values and the definition of intermediate results of serodiagnostic tests. J. Immunol. Met. 185, 123–132. doi: 10.1016/0022-1759(95)00121-P 7665894

[B12] Habibzadeh F. Habibzadeh P. Yadollahie M. (2016). On determining the most appropriate test cut-off value: The case of tests with continuous results. Biochemia Medica. 26, 297–307. doi: 10.11613/BM.2016.034 27812299 PMC5082211

[B13] Hickman S. E Woitek R. Le E. P. V. Im Y. R. Mouritsen Luxhøj C. Aviles-Rivero A. I. . (2022). Machine learning for workflow applications in screening mammography: systematic review and meta-analysis. Radiology 302, 88–104. doi: 10.1148/radiol.2021210391 34665034 PMC8717814

[B14] Hicks S. A. Strümke I. Thambawita V. Hammou M. Riegler M. A. Halvorsen P. . (2022). On evaluation metrics for medical applications of artificial intelligence. Sci. Rep. 12:5979. doi: 10.1038/s41598-022-09954-8 35395867 PMC8993826

[B15] Hovda T. Larsen M. Romundstad L. Sahlberg K. K. Hofvind S. . (2023). Breast cancer missed at screening; hindsight or mistakes? Eur. J. Radiol. 165, 1–6. doi: 10.1016/j.ejrad.2023.110913 37311339

[B16] Huang J. Chan P. S. Lok V. Chen X. Ding H. Jin Y. . (2021). Global incidence and mortality of breast cancer: a trend analysis, Aging 13, 5748–5803. doi: 10.18632/aging.202502 33592581 PMC7950292

[B17] Ignatyeva V. I. Kontsevaya A. V. Kalinina A. M. Drozdova L. Y. Mukaneeva D. K. Drapkina O. M. (2024). Socio-economic efficiency of the early cancer detection during the medical checkup. Russian J. Prevent. Med. 27, 36–44. (In Russ.). doi: 10.17116/profmed20242701136

[B18] Kim H. Yoon T. I. Kim S. Lee S. B. Kim J. Chung I. Y. (2023). Age-Related Incidence and Peak Occurrence of Contralateral Breast Cancer, JAMA Net. Open 6:e2347511. doi: 10.1001/jamanetworkopen.2023.47511 38100108 PMC10724757

[B19] Kim H. E. Kim H. H. Han B. K. Kim K. H. Han K. Nam H. . (2020). Changes in cancer detection and false-positive recall in mammography using artificial intelligence: a retrospective, multireader study. Lancet Digital Health 2, e138–e148. doi: 10.1016/S2589-7500(20)30003-0 33334578

[B20] Kudryavtsev N. D. Kozhikhina D. D. Goncharova I. V. Shulkin I. M. Sharova D.E. . (2024). The impact of artificial intelligence on double reading of mammograms. Profilakticheskaya meditsina 25:32. doi: 10.17116/profmed20242705132

[B21] Kumar R. Indrayan A. (2011). Receiver operating characteristic (ROC). curve for medical researchers. Indian Pediat. 48, 277–287. doi: 10.1007/s13312-011-0055-4 21532099

[B22] Le E. P. V. Wang Y. Huang Y. Hickman S. Gilbert F. J. (2019). Artificial intelligence in breast imaging, Clin. Radiol. 74, 357–366. doi: 10.1016/j.crad.2019.02.006 30898381

[B23] Liu J. Lei J. Ou Y. Zhao Y. Tuo X. Zhang B. . (2022). Mammography diagnosis of breast cancer screening through machine learning: a systematic review and meta-analysis. Clin. Exp. Med. 23, 2341–2356. doi: 10.1007/s10238-022-00895-0 36242643

[B24] Liu P. R. Lu L. Zhang J. Y. Huo T. T. Liu S. X. Ye Z. W. (2021). Application of artificial intelligence in medicine: an overview, Curr. Med. Sci. 41, 1105–1115. doi: 10.1007/s11596-021-2474-3 34874486 PMC8648557

[B25] Liu Y. Chen P. C. Krause J. Peng L. (2019). How to read articles that use machine learning, JAMA 322:1806. doi: 10.1001/jama.2019.16489 31714992

[B26] Martínez-Camblor P. Pardo-Fernández J. C. (2019). The youden index in the generalized receiver operating characteristic curve context. Int. J. Biostatist. 15, 1–20. doi: 10.1515/ijb-2018-0060 30943172

[B27] Morozov S. P. Andreychenko A. E. Kirpichev Y. S. Pavlov N. A. Meshalkin Y. E. Kokina D. Y. (2020a). Certificate of state registration of the PC program No. 2020664321 Russian Federation. MedLabel – automated analysis of medical protocols. Russia: State Budgetary Healthcare Institution of the City of Moscow Scientific and Practical Clinical Center for Diagnostics and Telemedicine Technologies of the Moscow Department of Health (GBUZ NPKTs DiT DZM).

[B28] Morozov S. P. Vetsheva N. N. Didenko V. V. Smirnov I. V. Ovsyannikov A. G. Ledihova N. V. . (2020b). Organization of a population screening program for the breast cancer among the female population: Methodological recommendations. Series Best Practices in Radiological and Instrumental Diagnostics. Moscow: Research and Practical Clinical Center for Diagnostics and Telemedicine Technologies of the Moscow Health are Department.

[B29] Morozov S. P. Vetsheva N. N. Ovsyannikov A. G. Ledihova N. V. Panina E. V. Polishchuk N. S. . (2019). Moscow screening: breast cancer screening with mammography as a method of improving early stage cancer detection. Problemy sotsial'noi gigieny, zdravookhraneniia i istorii meditsiny 27:629. 31747154 10.32687/0869-866X-2019-27-si1-623-629

[B30] Olczak J. Pavlopoulos J. Prijs J. Ijpma F. F. A. Doornberg J. N. Lundström C. . (2021). Presenting artificial intelligence, deep learning, and machine learning studies to clinicians and healthcare stakeholders: an introductory reference with a guideline and a Clinical AI Research (CAIR) checklist proposal. Acta Orthopaedica 92, 513–525. doi: 10.1080/17453674.2021.1918389 33988081 PMC8519529

[B31] Ovsyannikov A. G. Morozov S. P. Govorukhina V. G. Didenko V. V. Puchkova O. S. Pavlov N. A. . (2020). Prospect of application of artificial intelligence systems for breast cancer screening. Probl. Oncol. 66, 603–608. doi: 10.37469/0507-3758-2020-66-6-603-608

[B32] Pashayan N. Morris S. Gilbert F. J. Pharoah P. D. P. (2018). Cost-effectiveness and benefit-to-harm ratio of risk-stratified screening for breast cancer a life-table model. JAMA Oncol. 4, 1504–1510. doi: 10.1001/jamaoncol.2018.1901 29978189 PMC6230256

[B33] Paulden M. (2020). Calculating and interpreting ICERs and net benefit. PharmacoEconomics 38, 785–807. doi: 10.1007/s40273-020-00914-6 32390067

[B34] Pavlovich P. I. Bronov Y. Kapninsky A. A. Abovich Yu A. N. Rychagova I. . (2021). Comparative study of the digital mammography data analysis system based on artificial intelligence ‘Celsus' and radiologists. Digital Diagnost. 2, 22–23. doi: 10.17816/DD83184

[B35] Peirce C. S. (1884). The numerical measure of the success of predictions. Science 4, 453–454. doi: 10.1126/science.ns-4.93.453-a 17795531

[B36] Pencina M. J. Agostino R. B. D. Agostino R. B. D. Vasan R. S. (2008). Evaluating the added predictive ability of a new marker: From area under the ROC curve to reclassification and beyond. Statist. Med. 27, 157–172. doi: 10.1002/sim.2929 17569110

[B37] Pfohl S. Xu Y. Foryciarz A. Ignatiadis N. Genkins J. Shah N. H. (2022). Net benefit, calibration, threshold selection, and training objectives for algorithmic fairness in healthcare, in 2022 ACM Conference on Fairness, Accountability, and Transparency. New York, NY, USA: ACM 1039–1052. doi: 10.1145/3531146.3533166

[B38] Radice D. Redaelli A. (2003). Breast cancer management: quality-of-life and cost considerations. Pharmacoeconomics 384–396. doi: 10.2165/00019053-200321060-00003 12678566

[B39] Rousson V. Zumbrunn T. (2011). Decision curve analysis revisited: overall net benefit, relationships to ROC curve analysis, and application to case-control studies. BMC Med. Informat. Dec. Making 11:45. doi: 10.1186/1472-6947-11-45 21696604 PMC3148204

[B40] Salim M. Wåhlin E. Dembrower K. Azavedo E. Foukakis T. Liu Y. . (2020). External evaluation of 3 commercial artificial intelligence algorithms for independent assessment of screening mammograms. JAMA Oncol. 6:1581. doi: 10.1001/jamaoncol.2020.3321 32852536 PMC7453345

[B41] Seely J. M. Alhassan T. (2018). Screening for breast cancer in 2018—what should we be doing today? Curr. Oncol. 25, 115–124. doi: 10.3747/co.25.3770 29910654 PMC6001765

[B42] Singh K. Shah N. H. Vickers A. J. (2023). Assessing the net benefit of machine learning models in the presence of resource constraints. JAMIA 30, 668–673. doi: 10.1093/jamia/ocad006 36810659 PMC10018264

[B43] Taylor-Phillips S. Stinton C. (2020). Double reading in breast cancer screening: considerations for policy-making, Br. J. Radiol. 93:20190610. doi: 10.1259/bjr.20190610 31617741 PMC7055445

[B44] Vasilev Y. A. Rumyantsev D. Vladzymyrskyy A. Omelyanskaya O. Pestrenin L Shulkin I. . (2025). Evolution of an artificial intelligence-powered application for mammography. Diagnostics 15:822. doi: 10.3390/diagnostics15070822 40218172 PMC11988740

[B45] Vasilev Y. A. Rumyantsev D. Vladzymyrskyy A. Omelyanskaya O. Pestrenin L. Shulkin I. . (2023c). The first 10,000 mammography exams performed as part of the ‘Description and interpretation of mammography data using artificial intelligence' service. Manager Zdravookhranenia 54–67. doi: 10.21045/1811-0185-2023-8-54-67

[B46] Vasilev Y. A. Sychev D. A. Bazhin A. V. Shulkin I. M. Vladimirsky A. V. Golikova A. . (2024). Autonomous artificial intelligence for sorting the preventive imaging studies' results. Russian J. Prevent. Med. 27. 23–29. doi: 10.17116/profmed20242707123

[B47] Vasilev Y. A. Vladzymyrskyy A. Arzamasov K. Omelyanskaya O. Shulkin I. Kozikhina D. . (2023b). Clinical application of radiological AI for pulmonary nodule evaluation: Replicability and susceptibility to the population shift caused by the COVID-19 pandemic. Int. J. Med. Informat. 178:105190. doi: 10.1016/j.ijmedinf.2023.105190 37603940

[B48] Vasilev Y. A. Vladzymyrskyy A. V. Arzamasov K. M. Shulkin I. M. Kozhikhina D. D. (2023a). Double-reading mammograms using artificial intelligence technologies: A new model of mass preventive examination organization. Digital Diagnost. 4, 93–104. doi: 10.17816/DD321423

[B49] Vickers A. J. Van Calster B. Steyerberg E. W. (2016). Net benefit approaches to the evaluation of prediction models, molecular markers, and diagnostic tests. BMJ 352:i6. doi: 10.1136/bmj.i6 26810254 PMC4724785

[B50] Vickers A. J. van Calster B. Steyerberg E. W. (2019). A simple, step-by-step guide to interpreting decision curve analysis. Diagnost. Prognost. Res. 3, 1–8. doi: 10.1186/s41512-019-0064-7 31592444 PMC6777022

[B51] Wang J. Greuter M. J. W. Zheng S. van Veldhuizen D. W. A. Vermeulen K. M. Wang Y. . (2022). Assessment of the benefits and cost-effectiveness of population-based breast cancer screening in urban China: a model-based analysis. Int. J. Health Policy Manag. 11, 1658–1667. doi: 10.34172/ijhpm.2021.62 34273933 PMC9808213

[B52] World Health Organization (2022). A short guide to cancer screening. Increase effectiveness, maximize benefits and minimize harm. Copenhagen.

[B53] Youden W. J. (1950). Index for rating diagnostic tests. Cancer 3, 32–35. doi: 10.1002/1097-0142(1950)3:1<32::aid-cncr2820030106>3.0.co;2-3 15405679

